# Supplementary figures and images for: Mechanical Characterization of Dissolving Microneedles: Factors Affecting Physical Strength of Needles
Source: Pharmaceutics. 2024 Jan 30;16(2):200. doi: 10.3390/pharmaceutics16020200 (PMC10893124; doi:10.3390/pharmaceutics16020200)

(A)

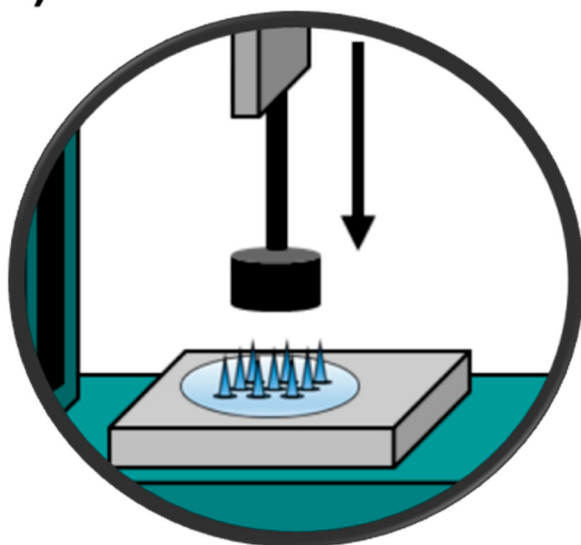

(B)

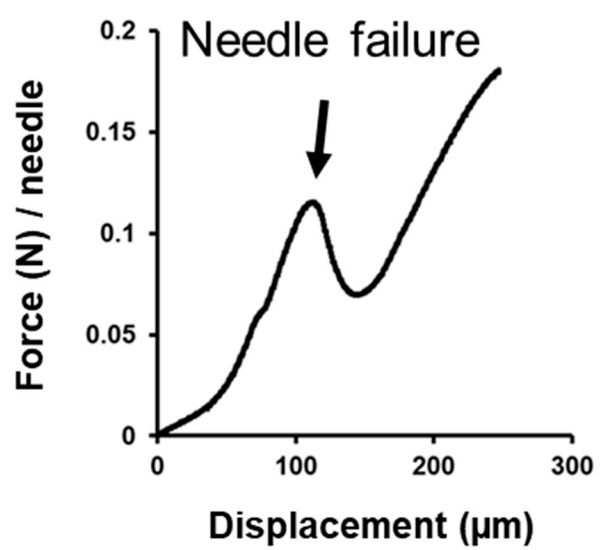

(C)

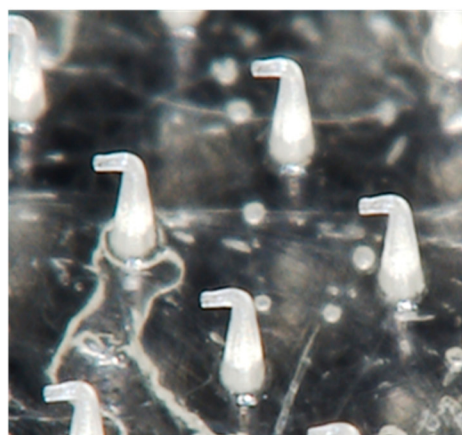

Supplement: Supplementary file 1 [file pharmaceutics-16-00200-s001.zip › Figure S1.pdf]
